# A multi-reference poly-conformational method for *in silico* design, optimization, and repositioning of pharmaceutical compounds illustrated for selected SARS-CoV-2 ligands

**DOI:** 10.7717/peerj.14252

**Published:** 2022-11-24

**Authors:** Vadim Alexandrov, Alexander Kirpich, Omar Kantidze, Yuriy Gankin

**Affiliations:** 1 Liquid Algo LLC, Hopewell Junction, NY, United States of America; 2 Department of Population Health Sciences, School of Public Health, Georgia State University, Atlanta, GA, United States of America; 3 Quantori, Cambridge, MA, United States of America

**Keywords:** Conformers, Multi-reference, Poly-conformational, SARSCOV- 2, *In silico*, Ligand-based, Structure-based, Virtual library, Computational framework, Validation

## Abstract

**Background:**

This work presents a novel computational multi-reference poly-conformational algorithm for design, optimization, and repositioning of pharmaceutical compounds.

**Methods:**

The algorithm searches for candidates by comparing similarities between conformers of the same compound and identifies target compounds, whose conformers are collectively close to the conformers of each compound in the reference set. Reference compounds may possess highly variable MoAs, which directly, and simultaneously, shape the properties of target candidate compounds.

**Results:**

The algorithm functionality has been case study validated *in silico,* by scoring ChEMBL drugs against FDA-approved reference compounds that either have the highest predicted binding affinity to our chosen SARS-CoV-2 targets or are confirmed to be inhibiting such targets *in-vivo*. All our top scoring ChEMBL compounds also turned out to be either high-affinity ligands to the chosen targets (as confirmed in separate studies) or show significant efficacy, *in-vivo*, against those selected targets. In addition to method case study validation, *in silico* search for new compounds within two virtual libraries from the Enamine database is presented. The library’s virtual compounds have been compared to the same set of reference drugs that we used for case study validation: Olaparib, Tadalafil, Ergotamine and Remdesivir. The large reference set of four potential SARS-CoV-2 compounds has been selected, since no drug has been identified to be 100% effective against the virus so far, possibly because each candidate drug was targeting only one, particular MoA. The goal here was to introduce a new methodology for identifying potential candidate(s) that cover multiple MoA-s presented within a set of reference compounds.

## Introduction

Most of the small molecules exist in multiple conformations (shapes) based on their surrounding environmental conditions. Each 3D shape of a molecule enables it to fit into the binding pockets of proteins and dictates its biological activity. Often, distinctly different chemical compounds that have similar shapes and similar charge distributions along the molecular surface can bind the same target. Therefore, it is beneficial to compare shapes and surface distribution charges for target query and reference compounds on a conformer-by-conformer basis. If one of the conformers of the query molecule matches one of the conformers (especially bound-to-target) of the reference molecule, there is a chance that the reference compound will also exhibit similar binding properties to the same target.

OpenEye Scientific Software Inc. pioneered an algorithm and the corresponding tool, Rapid Overlay of Chemical Structures (ROCS) (Grant, Gallardo & Pickup, 1996; OpenEye Scientific Software, Inc., 2008) for comparing shapes of molecules by overlaying and measuring their molecular structures *in silico* and comparing differences between a query and reference molecule. ROCS performs a shape-based overlay of a query conformer to a potential hit molecule by utilizing Gaussian atom-centered functions. The overlap is then expressed as a normalized value ranging between “0” and “1”, where “0” means no overlay and “1” means the maximum possible overlay. Atomic charges on the surface of the molecule are accounted for by using a separate score called “color”, so ROCS algorithm attempts to maximize both shape and “color” overlay. The “color” score also ranges from “0” to “1”, where “0” means no “color” similarity and “1” means a perfect color overlay. The final score thus ranges from 0 to 2. Thus, ROCS identifies potentially active compounds by comparing their shapes via *explicit alignment*; it is competitive and often superior to structure-based approaches in virtual screening (Hawkins, Skillman & Nicholls, 2007; Venhorst et al., 2008) both in terms of overall performance and consistency (Sheridan, McGaughey & Cornell, 2008). As a result, novel molecular scaffolds have been identified by using ROCS against various targets which have been considered very difficult to address computationally (Kumar et al., 2014; Kumar et al., 2016; Chen et al., 2018; Khan et al., 2019; Rush 3rd et al., 2005).

Being a computationally-intensive process, the overlapping of molecular shapes represents a bottleneck in the search for similar molecules. This remains despite the recent so-called PAPER implementation of ROCS on GPU (Haque & Pande, 2010) and the development of FastROCS (OpenEye Scientific Software, Inc, 2011) for large (>1B) compound libraries. Alternative methods perform overlaying by comparing shape-based descriptors *without* performing explicit shape alignment, specifically conformer-level 3D fingerprints. An example of such an approach is ElectroShape, implemented in the the Open Drug Discovery Toolkit (ODDT) package (Wójcikowski et al., 2019) which uses an algorithm incorporating shape, chirality, and electrostatics (Ballester & Richards, 2007b; Armstrong et al., 2010) and represents each conformer via a fixed-length vector of real-valued numbers. Similarly, the Extended 3-Dimensional FingerPrint (E3FP) package (Axen et al., 2017) also utilizes an *alignment-invariant* 3D representation of molecular conformers as a fixed-length binary vector for each conformer. These fingerprint-based approaches allow for the similarity calculation between two molecular shapes either as a Tanimoto distance (for binary fingerprints) or Euclidean distance (for real-valued fingerprints) computations. Such computations are orders of magnitude faster in comparison to alternative methods that require the actual alignment of the two compared conformers. The Ultrafast Shape Recognition (USR) method can speed up such virtual screening even more (Ballester & Richards, 2007a). Although the calculation of a shape-based fingerprint for each conformer can be a rather computationally involved procedure, as soon as all conformers for the virtual library are fingerprinted and stored in a database, the similarity search for the query molecule in such a database is computationally quick. Comparative performance of the two approaches (explicit shapes alignment *vs* shapes’ fingerprints comparison) in terms of accuracy (hit enrichment) was exhaustively studied for many virtual screening setups (Hawkins, Skillman & Nicholls, 2007; Kirchmair et al., 2009; Schreyer & Blundell, 2012) and was found to be comparable.

Here, we present MultiRef3D, a novel computational multi-reference poly-conformational algorithm for the design, optimization, and repositioning of pharmaceutical compounds. The algorithm searches for small molecules by comparing similarities between conformers of the same compound and identifies hits, whose conformers are collectively close to the conformers of each compound in a reference set. Reference compounds may represent well-characterized ligands and possess a highly variable mode of action (MoA). The principal and computationally efficient feasibility of this task is illustrated here by using pharmaceuticals that have been shown *in silico* to bind three different proteins of the SARS-CoV-2 as reference compounds. Although the SARS-CoV-2-induced COVID-19 pandemic is one of the biggest challenges worldwide, the highly effective drug for SARS-CoV-2 treatment has not been developed yet. Thus, the potential identification of the small molecule simultaneously targeting several viral proteins may represent an efficient antiviral drug discovery strategy. Moreover, for such a multi-reference search viruses which are the biological systems strongly dependent on the activity of different proteins may represent more illustrative examples than complex age-associated diseases such as cancer, neurodegeneration, psychiatric disorders, *etc*.

For method case study validation, we used the public ChEMBL (version 28) database (Gaulton et al., 2011) to screen compounds against the most important viral targets, namely 3C-like protease (3CLpro; Mpro), papain-like protease (PLpro) and RNA-dependent RNA polymerase (RdRp). These targets play a major role in virus replication/transcription and host cell recognition and are, therefore, vital for the viral reproduction and spread of infection. Since the method doesn’t directly use target information but rather analyzes 3D shapes for a compound that was already predicted, or has been experimentally found to be effective against a particular target (a reference compound), one has to choose one (or more) such compounds as a reference for each target. The focus for each of the above SARS-CoV-2 targets (3CLpro, PLpro and RdRp) was on the reference compounds with the highest binding affinities from the recent *in silico* multi-target repurposing study (Murugan et al., 2020). For the new compound search (virtual library screening) we used the same set of reference compounds as we used for the method case study validation.

## Materials & Methods

### Representative conformer space and conformer-by-conformer comparison

The proposed computational algorithm extends upon currently available methods (Ballester & Richards, 2007b; Armstrong et al., 2010; Axen et al., 2017; Wójcikowski et al., 2019) and introduces additional search flexibility via the use of the compound conformers. The proposal is to compare multiple possible shapes, adopted via varying environmental conditions, of the same molecule (*i.e.*, conformers) rather than just a single shape that was used previously. In particular, the suggested approach is based on matching ligand-ligand fingerprints without explicitly using target structure information, in contrast to docking and molecular dynamics approaches that simulate the physical binding of a ligand to the target. The supporting theory behind the method is based on the decision to treat conformers, which might have different binding characteristics and properties, as independent entities. In such an approach, each conformer has the corresponding independent alignment-free, 3D-similarity scoring using known multi-references. All conformers were generated using the Experimental-Torsion basic Knowledge Distance Geometry (ETKDG) algorithm implemented in RDkit (Wang et al., 2020b). ETKDG builds on the classical Blaney and Dixon’s (Blaney & Scott Dixon, 2007) Distance Geometry (DG) algorithm (sampling from all theoretically possible interatomic distances in a given molecule) by combining knowledge of preferred Torsional angles derived from Experimentally determined crystal structures (ETDG), and by further adding constraints from chemical Knowledge, such as ‘aromatic rings are flat’, or ‘bonds connected to triple bonds are colinear’ (ETKDG).

Benchmarking studies have found ETKDG to be the best-performing freely available conformer generator up-to-date (Friedrich et al., 2017b; Friedrich et al., 2017a) providing diverse and chemically-meaningful conformers reproducing crystal conformations.

Unlike what the majority of computational methods had assumed a couple of decades ago (*e.g.* in the CoMFA method (Gohda et al., 2000)), recent research indicates that the bioactive conformation is not necessarily the lowest-energy conformation in the presence of the receptor (Hasegawa, Arakawa & Funatsu, 2000; Mackerell Jr, 2004; Acharya et al., 2011). In particular, as long as an increase in energy for less favorable conformation is compensated by its binding to the target, *i.e.* the total ligand-target energy is lower than the sum of the energies for the non-bound target and ligand, the bound state is favored. The proposed method emphasizes and relies on this ligand’s ability to use its potentially higher energy conformations, depending on the target it attempts to bind. Note, however, that when a sufficiently large number of conformers is requested, ETKDG algorithm generates more conformers with lower energy than with higher energy (Friedrich et al., 2017b; Friedrich et al., 2017a), therefore when averaged over all conformers (and we generate 100 conformers per molecule), conformers with the lower energy will contribute more to the total overlap.

One of the things that distinguishes ligand-based 3D virtual screening methods from 2D methods is that one has to start worrying about how many conformers to include in the reference set. If the molecule is flexible, it can assume many shapes and pharmacophores. How to deal with this is one of the fundamental questions in ligand-based virtual screening (LBVS).

In a recent paper by the Schrödinger team, Cappel et al. (2014) performed comprehensive benchmark analysis and found that the number of conformers needed for 3D LBVS is actually relatively low: 100 or less to achieve good performance. Thus, we used ETKDG to generate 100 conformers per molecule in this work compound to make sure that the conformational space is adequately covered. Some query ligands with few rotatable bonds can have many very similar conformers, which would mean that the molecule “spends more time” in such conformational states, which would further mean that if those conformers overlap closely with the reference conformers, the result would be more statistically sound (the contributions of such “overrepresented” conformers would weigh more in the average query-reference overlap score for that query compound, in the spirit of Gibbs sampling (Geman & Geman, 1984; Seep et al., 2021), which assigns greater weight to samples that occur more frequently in the sampled distribution.

**Figure 1:**
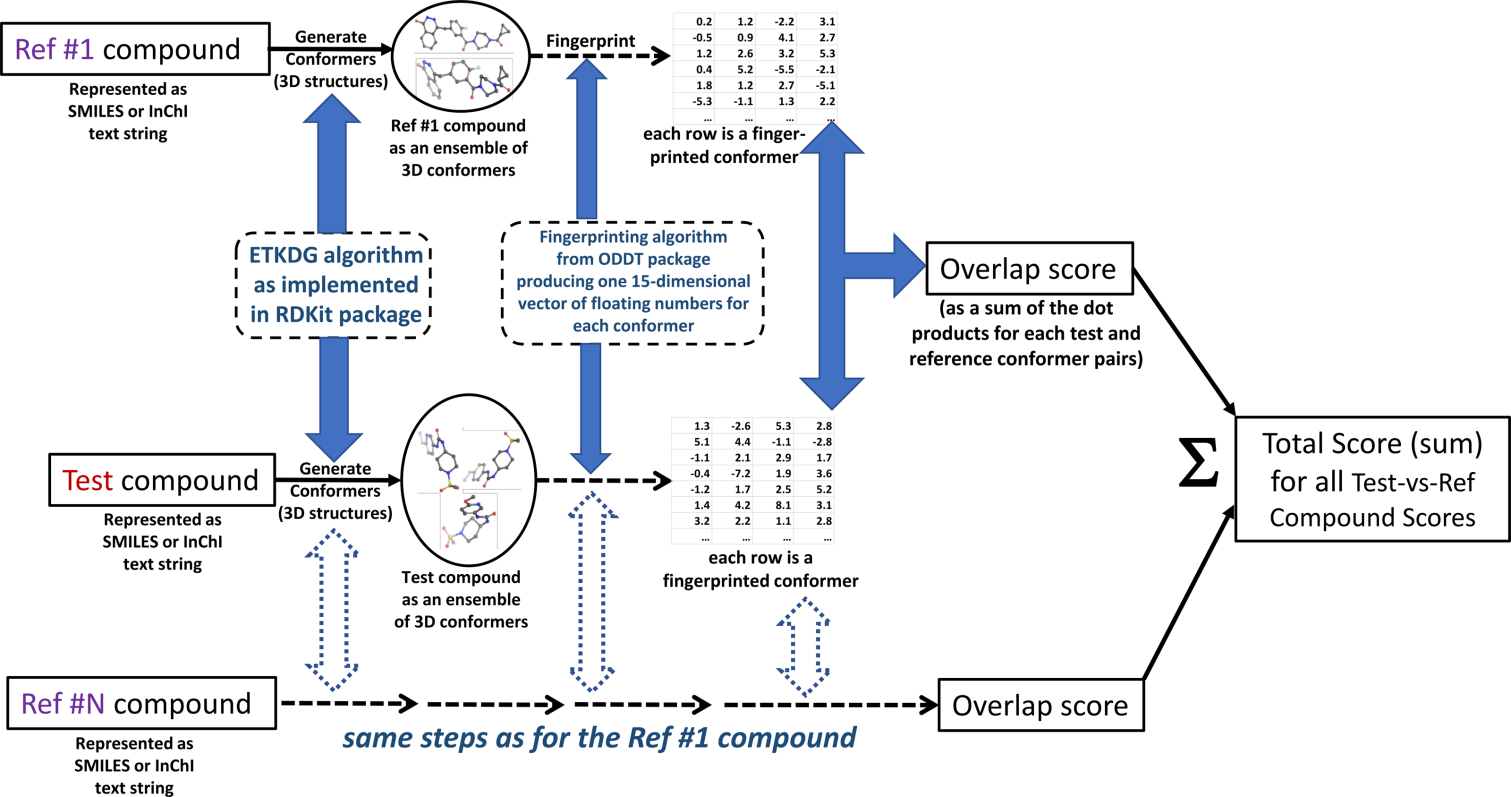
MultiRef3D screening method diagram for multi-conformer and multi-reference screening procedure. For each test compound multiple conformers and the corresponding overlapping scores are computed. Later, the overlapping scores are summed into the total score for the selected test compound. The figure has been created in Microsoft PowerPoint 2016, pyMOL v2.5 (pymol.org) and RDKit v2021.03.01 software (rdkit.org).

The authors call the approach MultiRef3D to emphasize that it is a fast, alignment-free multi-objective optimization protocol that maximizes the 3D overlap of a query molecule’s conformational ensemble with conformational ensembles of multiple reference ligands. The diagram of the proposed method is summarized in Fig. 1. The formal details of the approach are discussed further.

### Efficiency and a conformer scoring

Fingerprinting of individual conformers for alignment-free comparisons became popular in the past few years (Wójcikowski, Zielenkiewicz & Siedlecki, 2015; Axen et al., 2017; Gladysz et al., 2018; Wang et al., 2020a). In our algorithm, each conformation is treated as an independent entity and is characterized by a vector of features (fingerprint) which describes its 3D shape along with the distribution of electrostatic charge (both denoted further as electroshape) across its molecular surface. In this work, we used 15-dimensional USRCAT fingerprints (Schreyer & Blundell, 2012) which distil molecular shape into a rotation-invariant descriptor vector made up of 15 real numbers describing distance distribution among atoms, atomic partial charges, and atom types. The USRCAT fingerprints were shown to significantly outperform just shape-based fingerprints in recent benchmark tests (Schreyer & Blundell, 2012; Bonanno & Ebejer, 2020). Since USRCAT fingerprints reflect both relative 3D positions for all atom types and molecular surface charges for each query molecule conformer as well as for all conformers of the reference compound, they are very well-suited for alignment-free fast computation of conformer similarity. Each conformer is coded within the algorithm by a single fingerprint represented as a fixed-length vector of numbers which ensures computational efficiency. These fingerprints for each of the query and reference molecule conformers are individually scored by the Euclidean distance, serving as a similarity measure between two conformers. Note that both Euclidean distance and Tanimoto index can serve as similarity measures for these and other real-valued fingerprints (Bajusz, Rácz & Héberger, 2015). We chose Euclidean distance as our metric since this choice for the shape-based fingerprints has been already extensively studied and validated, showing superior performance on the DUD dataset (Armstrong et al., 2010).

### Objective function optimization

The sum of the conformer-to-conformer similarity scores between the query and reference compound are compared via an objective similarity function *W*_*c*_ for each reference compound *c*. The goal is to maximize the sum of those individual objective similarity functions across all reference compounds of interest *c* = 1, 2, ..., *C* where *c* is a summation index for the desired set of reference compounds:

(1) \begin{eqnarray*}{W}_{All}={\mathop{\sum \nolimits }\nolimits }_{c=1}^{C}{W}_{c}={\mathop{\sum \nolimits }\nolimits }_{c=1}^{C}{\mathop{\sum \nolimits }\nolimits }_{q=1}^{Q}{\mathop{\sum \nolimits }\nolimits }_{r=1}^{R}{S}_{q,r.}^{(c)}.\end{eqnarray*}

In formula Eq. (1) the summand ${S}_{q,r}^{(c)}$ is the similarity (overlap) of the query conformer *q* (*q* = 1, 2, ..., *Q*) with the conformer *r* (*r* = 1, 2, ..., *R*) for each reference compound *c* (*c* = 1, 2, ..., *C*). For the real-valued fingerprints, the similarity summand between the pair of conformers of interest indexed by query index *q* and reference index *r* for compound *c* is calculated as:

(2) \begin{eqnarray*}{S}_{q,r}^{(c)}=1-(1/N)\sqrt{{\mathop{\sum \nolimits }\nolimits }_{n=1}^{N}({x}_{q,n}^{(c)}-{x}_{r,n}^{(c)})^{2}}\end{eqnarray*}

where ${x}_{q,n}^{(c)}$ and ${x}_{r,n}^{(c)}~$ are the corresponding normalized fingerprint vector coordinates for *n* = 1, 2, ..., *N*. The length (the number of coordinates) of the fingerprint *N* is determined based on the problem-specific target-ligand interaction characteristics. Since the fingerprint coordinates ${x}_{q,n}^{(c)}$ and ${x}_{r,n}^{(c)}$ are normalized (*i.e.* have values between 0 and 1 for each coordinate *n*) the resulting overlap ${S}_{q,r}^{(c)}$ is maximized with the value equal to 1, when the fingerprints of both conformers are identical, and can take the smallest value equal to 0, when all the fingerprint coordinates have a difference equal to 1 *i.e.* as different as possible at the normalized scale.

When the objective is to identify a novel compound for just a single active conformation (*r* = 1) of one (*c* = 1) reference compound (*e.g.* a reference ligand co-crystallized with one particular target) then all conformers for the query molecule are scored against only one active reference conformer. However, in the case when multiple reference compounds are bound to the same target (or sets of reference compounds bound to multiple targets), the total objective function comes into play. It is important to point out that the proposed method is not limited to the structure-based design situations: when several reference compounds are found to be active in a functional assay (and either the target(s) is unknown or the crystal structure of the target is not available)—the formula works just as well (as long as the ligand structure is known). The method becomes especially handy, when there is a great diversity among active reference compounds, whether the target structural information is known or not—the objective function will extract and sum up the similarities for all of the relevant parts of the fingerprinted conformer representations responsible for the observed activity.

The query compound can be evaluated against multiple reference compounds on a conformer-by-conformer basis. In such cases, the corresponding similarity scores are summed and constitute the multi-reference conformer-level objective function to maximize. This can be readily used in a typical ligand-based design setting. However, instead of just searching for a shape analogue of one of the conformers of a reference compound, in the case of multiple references, the algorithm performs a search for a compound in the virtual library whose conformers have overlapped with conformers of each of the reference compounds. The latter will increase the chances that the selected virtual compound binds the same way to the corresponding targets of each of the references (*i.e.* the selected compound is capable of forming conformations that resemble active conformations responsible for the MoA of each of the references).

Performance-wise, in comparison with explicit 3D alignment methods, MultiRef3D exhibits speed-ups closely resembling those achieved by USR-CAT (which is at the core of the MultiRef3D methodology) and scales linearly with the number of reference compounds in the query. Also, since the number of top hits for each reference (typically in the range 1,000–10,000) is set to be orders of magnitude lower than the number of compounds in the screening universe, the final step (hits scores summation and sorting) takes less than a millisecond on any modern single CPU (*e.g.* Intel Core i7). The comparative performance in relative units are summarized and presented in Table 1.

**Table 1:** The comparative performance of hit identification via different methods with MultiRef3D search time presented in relative time units. The search setup is identical to that described in Ballester & Richards (2007a).

**Method**	**Performance (Relative Time Units)**
MultiRef3D	1
EShape3D	515
Shape signatures	679
ROCS	4745

### Method case study validation for known targets

To case study validate the proposed methodology for the multi-target-specific conformer similarity the three following targets have been used: 3CLpro (Mpro), PLpro, and RdRp of the SARS-CoV-2. The spike protein has not been included as the validation target since the pharmacological activity may not be correlated directly with the binding affinity to the interfacial site (Murugan et al., 2020). ChEMBL (version 28) public database (Gaulton et al., 2011) has been chosen as the universe for screening. The selected ChEMBL compounds were already marketed drugs for which at least one target is known. The corresponding ChEMBL extraction query is provided in the code available on GitHub (https://github.com/quantori/MultiRef3D). The screened set had a total of 2,604 compounds. The corresponding reference compounds for case study validation were selected from the recent multi-target *in silico* repurposing study (Murugan et al., 2020) based on the highest binding affinities for each of the targets.

### Compounds search based on the conformers of the reference compounds

One hundred conformers for each of the reference molecules were generated at the MMFF94 (Halgren, 1996) and each conformer was ODDT-fingerprinted (Wójcikowski et al., 2019) and saved in the MongoDB database (MongoDB, 2020). The ODDT implementation (Wójcikowski et al., 2019) of ElectroShape fingerprints (Armstrong et al., 2010) has been selected to demonstrate the proposed approach because these fingerprints are considered to be state-of-the-art in ligand-based virtual screening experiments (Cortés-Cabrera et al., 2013; Bonanno & Ebejer, 2020), and they are not limited to binary values.

### Virtual libraries for screening

Virtual libraries (query compounds) for screening consisted of an Enamine (Enamine, 2020) focused “antiviral-like” set (3995 compounds) and a diverse Discovery Diversity Set (10559 compounds) (Enamine, 2020). Molecules from each virtual library were simultaneously evaluated against several reference drugs with different MoA (3CLpro, PLpro and RdRp inhibition). A query molecule for which some of its conformers are similar in shape to conformers for all the reference drugs would receive a higher score. In this approach, multiple virtual compounds can be identified to have a good conformer overlap with the conformers of reference drugs.

## Results

### Method case study validation for SARS-CoV-2 Compounds

The highest affinity binder Olaparib (−9.2 kcal/mol) has been selected as a reference compound for 3CLpro, Tadalafil (−9.2 kcal/mol) for PLpro and Lumacaftor (−9.9 kcal/mol) for RdRp. However, when multi-target scoring against these three references has been performed, the top ten scoring compounds from ChEMBL *had no conformers* similar in 3D shape (Euclidean distance < 0.5) to Lumacaftor conformers. Therefore, the Lumacaftor reference has been replaced with the next best *in silico* RdRp binder Ergotamine (Murugan et al., 2020) (−9.4 kcal/mol). The resulting scores produced by the proposed method are summarized in Table 2.

**Table 2:** Top ten scoring compounds showing simultaneous conformer similarity with the reference compounds Olaparib, Tadalafil, and Ergotamine.

**Compound ID**	**Compound Name**	**TotalScore**	**Olaparib**	**Tadalafil**	**Ergotamine**
CHEMBL779	Tadalafil	228.46	70.70	100.00	57.76
CHEMBL1737	Sildenafil citrate	225.15	81.30	58.34	85.50
CHEMBL521686	Olaparib	223.08	100.00	57.61	65.48
CHEMBL105442	Ci-1040	220.40	80.68	79.16	60.56
CHEMBL129857	As-602868	220.16	78.27	74.50	67.39
CHEMBL2037511	Epelsiban	219.86	81.58	70.28	68.01
CHEMBL565612	Sotrastaurin	219.13	79.93	69.36	69.83
CHEMBL1516474	Tegaserod maleate	217.83	80.22	76.56	61.05
CHEMBL1236682	Refametinib	217.78	76.01	81.57	60.20
CHEMBL1923502	Ulimorelin hydrochloride	217.56	76.29	74.79	66.47

Both Olaparib and Tadalafil had the highest scores which confirmed the previous finding (Murugan et al., 2020) that these compounds are simultaneously good binders for *both* 3CLpro and PLpro. Our method has also picked up Sildenafil (brand name Viagra) which just like Tadalafil (Cialis) is also known as a classical PDE5A inhibitor. Although those compounds are predominantly used in the treatment of male erectile dysfunction and pulmonary hypertension, it was shown (Shirvaliloo, 2021) that in the presence of SARS-CoV-2 infection, PDE5 inhibitors prevent thromboembolism caused by inflammatory processes in COVID-19 patients via NO/cGMP pathway and are potent inhibitors of 3CLpro (Jin et al., 2020)

Ci-1040 and Refametinib are the other two hits from Table 2 and are potent MEK inhibitors with high 3D shape similarity to both Olaparib and Tadalafil. MEK inhibitors, including Olaparib (Vena et al., 2018) were recently demonstrated to reduce cellular expression of ACE2 while stimulating NK-mediated cytotoxicity and attenuating inflammatory cytokines during the severe stage of SARS-CoV-2 infection (Zhou et al., 2020). Ci-1040 was also previously shown to display a broad anti-influenza virus activity *in vitro* and to provide a prolonged treatment window compared to the standard of care *in vivo*, specifically in lung cells (Haasbach et al., 2017).

The other hit from Table 2 is Sotrastaurin, a PKC inhibitor that has been experimentally shown to inhibit SARS-CoV-2 replication *in vivo* (Liu et al., 2021) and found to be among the best 3CLpro binders during *in silico* ZINC database screening study (Olubiyi et al., 2020). The other top hit, Epelsiban, was originally developed as an oxytocin receptor agonist. However, it has been recently shown (Kim et al., 2015) that oxytocin plays a major role in the activation of NF-kB-mediated pathways. Interestingly, recent research has revealed (Hariharan et al., 2021) that Remdesivir, in addition to being a potent RdRp inhibitor (Gordon et al., 2020), is also reducing viral replication via NF-kB pathway. Therefore, this hit serves as an example of non-obvious 3D-shape-based drug repurposing idea generation linked to the relevant yet non-primary SARS-CoV-2 inhibiting mechanisms of reference compounds.

In our second case study validation experiment, we explored what happens if the RdRp reference compound Ergotamine is replaced with Remdesivir which, as was already mentioned, is not only a well-established RdRp inhibitor and computationally found to be a tight RdRp binder but also a cytokine storm attenuator that works via NF-kB pathway. We were interested if the algorithm would pick up NF-kB hits and other potential “chain terminators”. The resulting scores produced in the second scoring setup are summarized in Table 3.

**Table 3:** Top ten scoring compounds showing simultaneous conformer similarity with the reference compounds Olaparib, Tadalafil, and Remdesivir.

**Compound ID**	**Compound Name**	**TotalScore**	**Olaparib**	**Tadalafil**	**Remdesivir**
CHEMBL1694	Benazepril hydrochloride	180.82	66.67	64.26	49.89
CHEMBL515606	Cilazapril	180.61	64.56	61.56	54.50
CHEMBL495727	At-9283	179.03	68.17	56.15	54.71
CHEMBL2107495	Temafloxacin hydrochloride	178.94	67.15	55.78	56.01
CHEMBL1200779	Trovafloxacin mesylate	178.60	66.24	54.02	58.35
CHEMBL340978	Benoxaprofen	178.27	68.56	56.54	53.16
CHEMBL8	Ciprofloxacin	177.05	63.21	57.19	56.65
CHEMBL1200831	Spirapril hydrochloride	177.00	65.28	60.24	51.47
CHEMBL1201011	Quinapril hydrochloride	176.84	66.32	60.40	50.13
CHEMBL1168	Ramipril	176.54	65.32	63.26	47.96

For Olaparib, Tadalafil, and Remdesivir reference compounds, half of the top ten hits (Benoxaprofen, Ciprofloxacin, Spirapril hydrochloride, Quinapril hydrochloride and Ramipril) turned out to be ACE inhibitors and coagulation modifiers acting via NF-kB related pathways (Hernández-Presa et al., 1997; Burzynski et al., 2019)! In addition, all of them turned out to be also good binders of 3CLpro (Biembengut & Brasil de Souza, 2020). One can also notice that the individual scores for Remdesivir-only hits are *significantly* (>10%) lower than the corresponding scores for the other reference compounds, which can be readily explained by the fact that none of the CHEMBL hits was a nucleoside and thus cannot be incorporated into the replicated RNA similar to the way Remdesivir does (although we still pick up the NF-kB component from Remdesivir’s MoA).

The other hits were Temafloxacin and Trovafloxacin, predicted to be potent 3CLpro ligands (Gimeno et al., 2020) and experimentally shown to inhibit virus replication (Krieg et al., 2006; Mirmotalebioshi et al., 2021) and anti-inflammatory drugs Benoxaprofen and Ciproflaxin predicted to target 3CLpro (Marciniec et al., 2020; Zeyrek et al., 2021) as well.

An interesting multi-target Aurora/JAK inhibitor, compound At-9283, closes the list (Table 3). JAK inhibitors have promising therapeutic potential for SARS-CoV-2 treatment with their dual anti-inflammatory and anti-viral effects (Mehta et al., 2020). At-9283 has also been recently identified to reverse SARS-CoV-2 transcriptomic signature (O’Donovan et al., 2020) and due to its similarity to tipiracil 3D pharmacophore scaffold, also inhibits SARS-CoV-2 Nsp15 endoribonuclease (Kandwal & Fayne, 2020; Guedes et al., 2021) and targets 3CLpro (Mengist, Dilnessa & Jin, 2021; Baby et al., 2021).

In summary, the results of these case study validation experiments show that MultiRef3D can efficiently identify compounds whose conformers simultaneously mimic the conformers of three different small molecules. All identified high-score compounds represent drugs that have direct or indirect evidence to be effective in anti-COVID-19 treatment.

### Virtual library screening for multi-target SARS-CoV-2 compounds

The results from the focused (“antiviral-like”) and diverse (“Discovery Diversity Set”) sets are summarized in Tables 1 and 2 respectively. These are given here for illustrative purposes only, to demonstrate that the hits are indeed simultaneously aligned with the references. The algorithm visual summary is displayed in Fig. 1 for the *W*_*All*_ objective function. Tables S1 and S2 summarize the direct application results of the Enamine (Enamine, 2020) focused “antiviral-like” and “Diverse Discovery Set” virtual library screening. The first two columns of the Tables contain query compound IDs and their computed overlap scores. The rows are sorted according to the total sum overlap score displayed in the second column.

**Figure 2:**
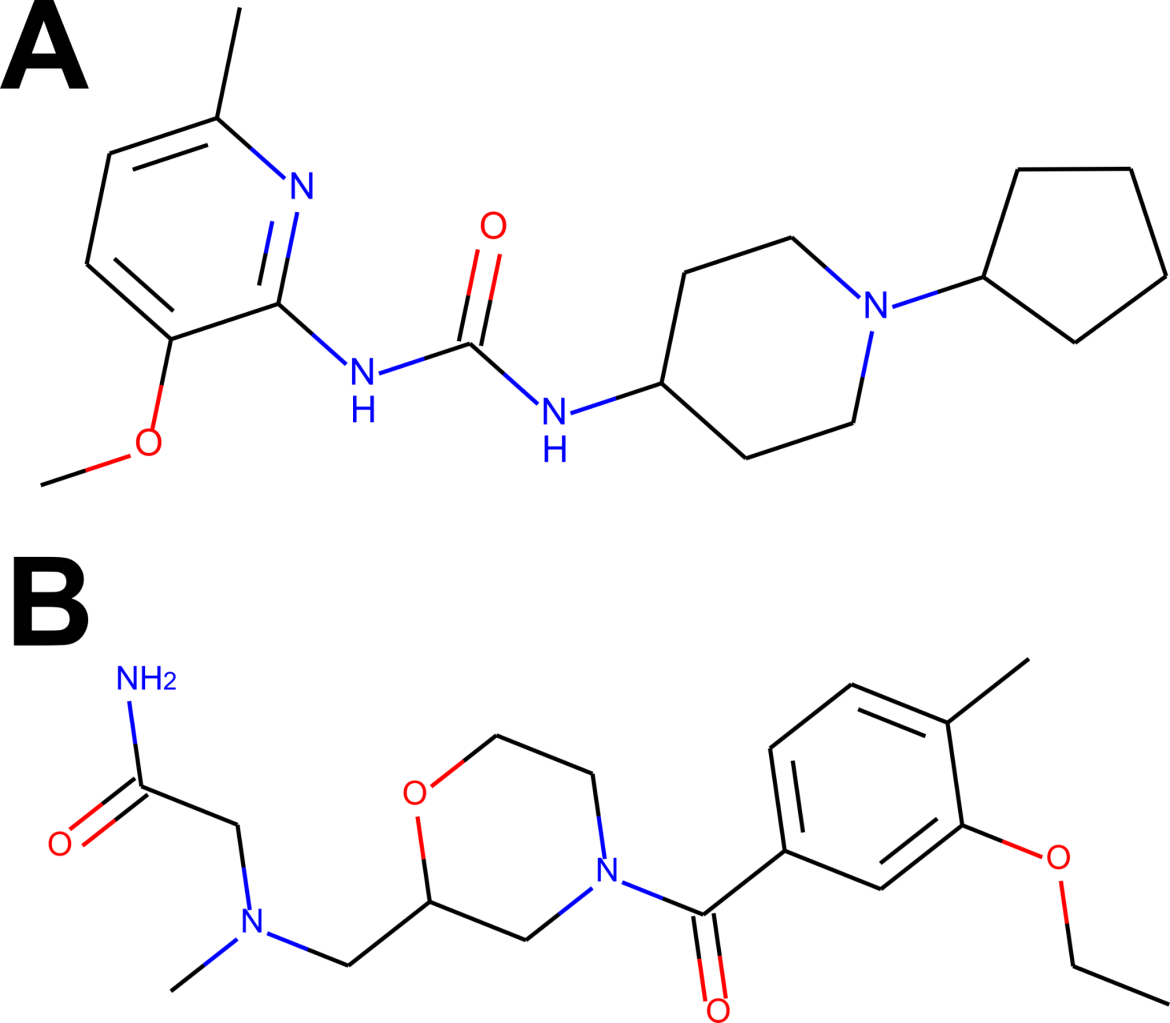
The compounds presented in panels A and B are the top hits Z1693453146 (*Wall* = 254.11) and ZZ1760146546 (*Wall* = 255.19) from the non-overlapping “antiviral-like” and “Discovery Diversity” libraries, respectively. One can immediately observe, however, that the compounds share a lot of similarity, in particular overall shape and amide bridge connecting heterocycles. The bridge allows for 3D flexibility for the molecule to change conformation and bind to multiple targets. The figure has been created in Microsoft PowerPoint 2016, pyMOL v2.5 (pymol.org) and RDKit v2021.03.01 software (rdkit.org).

**Figure 3:**
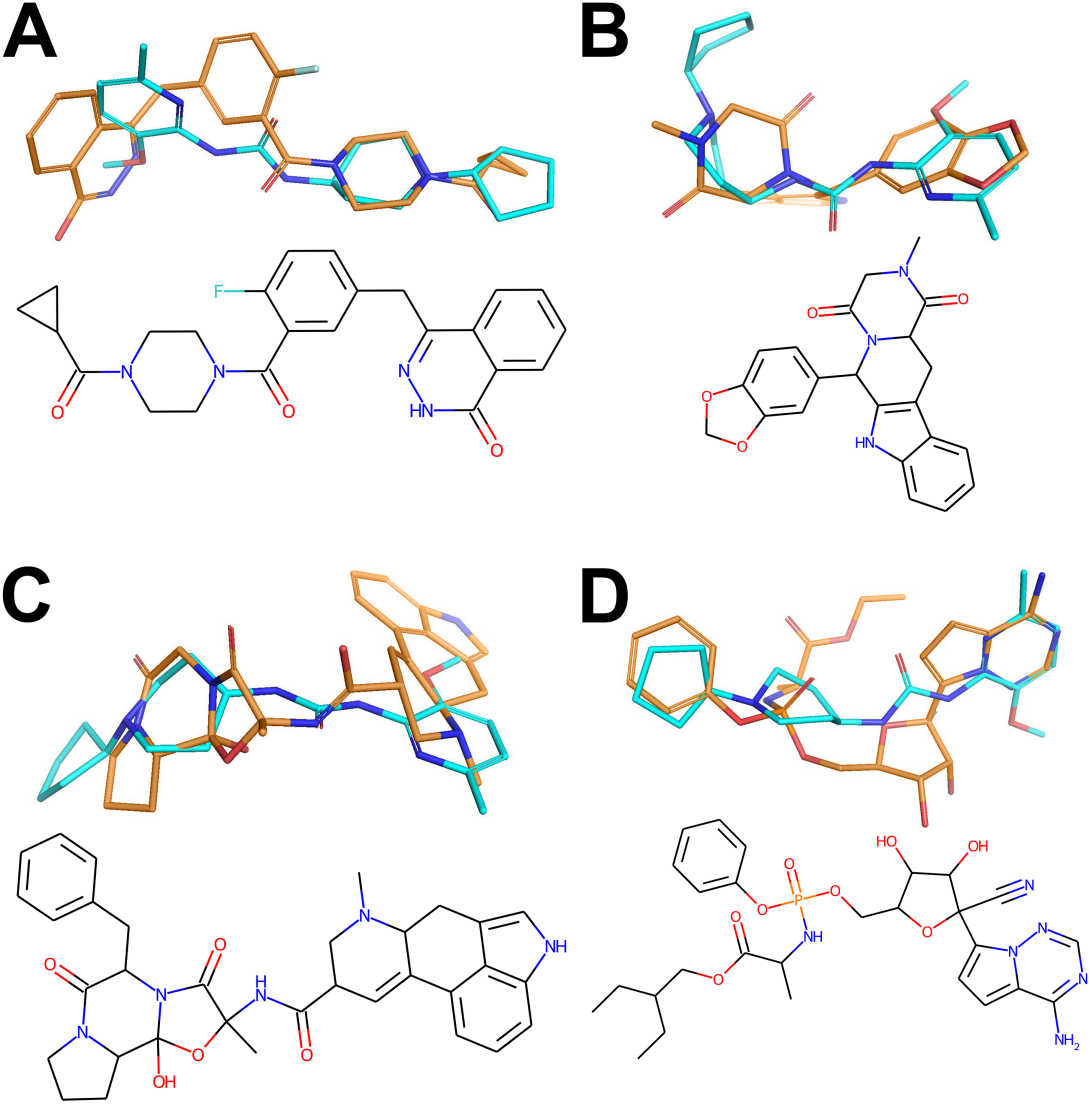
Active conformers of the Reference compounds (Olaparib, Tadalafil, Ergotamine and Remdesivir on A, B, C and D, respectively) aligned with the best matching conformers of the top hit Z1693453146 (Wall = 254.11). Carbon-carbon bonds for the reference compounds and the Top Hit are shown in gold and cyan respectively (C-N and C-O bonds are conventionally shown in blue and red). The figure has been created in Microsoft PowerPoint 2016, pyMOL v2.5 (pymol.org), RDKit v2021.03.01 software (rdkit.org) and Adobe Photoshop CC v20.0.6.

For the visual illustration of the algorithm results, the two compounds with the highest scores from Tables S1 and S2 have been presented in Fig. 2. It is worth noting that these compounds are quite flexible molecules due to their amide bridge around which the ring substructures can rotate, which ensures the ability of those molecules to accommodate different targets. One can also notice that the Remdesivir component scores are significantly lower in comparison to other references (while Ergotamine component remains high), reflective of the facts that, just as in CHEMBL case, none of the hits was a nucleoside in nature (see also Fig. 3D).

The best-matching conformers of the top hit Z1693453146 spatially align with the active conformation for each reference drug (Fig. 3). One can observe that most of hydrogen donors and acceptors from the top hit conformer and reference conformers are aligned very well, mimicking the interaction patterns with each target. At least partial spatial alignment of atom types is expected from the top hit conformers since atom types as well as their relative 3D positions is the essence of the USRCAT fingerprints (Schreyer & Blundell, 2012).

## Discussion

### Computation efficiency and availability of the method

The proposed method does not rely on laborious docking and molecular dynamics setup, especially in the multi-target case, where target preparation and choice of method *i.e.* direct docking to a fixed-coordinate target or Molecular Dynamics-based ensemble energy minimization are of utmost importance and require deep expertise. Fingerprint comparison is orders of magnitude faster and simpler; it only requires simple structural information in the form of either isomeric SMILES or InChI. The entire setup, which is presented in the Supplementary Information, can be universally used for any multi-target screening and optimization, whenever reference compounds for each of the targets are available. Naturally, further hit refinement (ADMETox, PK/PD, *etc*) is necessary if the screened universe is not limited to drugs with well-known safety profiles.

Depending on what is known about the indication or marketed drug of interest (targets, MoAs, other existing drugs for the same indication), the proposed methods (or a combination thereof) can be used to find other non-obvious molecules whose shape and surface electrostatic charge is similar to that of the marketed drug. The methods can also be used to search for the cumulative similarity to conformers of the multiple drugs used to treat this disease indication.

In the proposed approach multiple conformers of the query ligand have been compared with conformers from *multiple* reference compounds whose therapeutic effect of interest is achieved via different mechanisms of bindings to different targets, *e.g.* by inhibiting major proteases 3CLpro and PLpro (Ullrich & Nitsche, 2020) and RNA-dependent RNA polymerase (RdRp) (Elfiky, 2020; Li et al., 2003; Vincent et al., 2005). An “ideal drug” would contain conformers that resemble (as many as possible) conformers of all the reference drugs, thus increasing chances that the drug inhibits SARS-CoV-2 via multi-MoA routes and is more effective than each individual reference drug.

When the crystal structure of the target protein is known and the reference ligand is co-crystallized in its active conformation (structure-based design), we can use this information about the reference compound and evaluate the query molecules against only one, the active (co-crystallized), reference ligand conformation (*r* = *r*_*active*_) in formulas Eqs. (1) and (2). Confirmation by direct docking for the fingerprint-matched queries can be used to confirm the match.

Our methodology emphasizes the pursuit of candidate compounds that achieve the therapeutic effect (*e.g.* stops SARS-CoV-2 proliferation) by multiple MoA routes. A successful candidate compound would contain conformers targeting the three SARS-CoV-2 factors (3CLpro, PLpro, RdRpall) at the same time by increasing the chances that the compound would protect against SARS-CoV-2 much more effectively. Naturally, all successful candidates would need to be further screened and filtered for proper ADME-Tox and other drug-likeness properties. Binding to anti-targets, *e.g.* hERG, can be explicitly incorporated into this methodology by adding the corresponding terms (similarities to known hERG-binding ligands) to the overlap sum with a negative sign. Even though many computational methods exist to evaluate hERG in particular as well as other common tox liabilities, when an anti-target is very specific and less commonly known as “pure tox target” (*e.g.* undesired binding to D2 receptor for many modern CNS drugs), the explicit inclusion of similarity score to such anti-target with a negative sign can greatly streamline the overall drug optimization process.

## Conclusions

We have demonstrated and case study validated the usefulness of the multi-reference computationally efficient optimization approach in drug discovery screening and repurposing scenarios. The method represents each molecule as an ensemble of flexible conformers that would choose the best possible conformation for each presented target-binding opportunity. The application of this approach to SARS-CoV-2 produced several antiviral drug candidates that are designed to protect against SARS-CoV-2 by multiple mechanisms simultaneously.

##  Supplemental Information

10.7717/peerj.14252/supp-1 Supplemental Information 1 Supplementary Tables

## List of Abbreviations

ADME-Tox: Absorption, Distribution, Metabolism, Excretion and Toxicity
GPU: Graphics processing unit
CNS: Central nervous system
CoMFA: Comparative molecular field analysis
COVID-19: Coronavirus Disease of 2019
CPU: Central processing unit
hERG: Human Ether-a-go-go-related Gene
MoA(s): Mechanism of Action(s)
ODDT: Open Drug Discovery Toolkit
RNA: Ribonucleic acid
ROCS: Rapid overlay of chemical structures
SARS-CoV-2: Severe acute respiratory syndrome coronavirus 2
USR: Ultrafast Shape Recognition
WHO: World Health Organization

## Additional Information and Declarations

### Data Availability

The following information was supplied regarding data availability: The code used for analysis is available at the Quantori GitHub repository: https://github.com/quantori/MultiRef3D and via Zenodo DOI: https://doi.org/10.5281/zenodo.7261953. The data (ligand structures) from REAL focused libraries is available at the Enamine Ltd: https://enamine.net/compound-collections/real-compounds/real-database; https://doi.org/10.1002/ejoc.202101210.
