# Supplementary material for: A multi-reference poly-conformational method for in silico design, optimization, and repositioning of pharmaceutical compounds illustrated for selected SARS-CoV-2 ligands
Source: PeerJ. 2022 Nov 24;10:e14252. doi: 10.7717/peerj.14252 (PMC9701500; doi:10.7717/peerj.14252)
Supplement: Supplemental Information 1 [file peerj-10-14252-s001.pdf]

A multi-reference poly-conformational method for in silico design, optimization, and repositioning of pharmaceutical compounds illustrated for selected SARS-CoV-2 ligands.

Vadim Alexandrov[1], Alexander Kirpich[2] , Omar Kantidze[3], and Yuriy Gankin[*3]

1 Liquid Algo LLC, 85 Thistle Lane, Hopewell Junction, NY 12533, United States of America

2 Department of Population Health Sciences, School of Public Health, Georgia State University, Atlanta, GA, United States of America

3 Quantori LLC, 625 Mass Ave, Cambridge, MA 02139, United States of America

*** Corresponding author**

E-mail: yuriy.gankin@quantori.com

SUPPLEMENT

Tab. S1. The top scoring compounds from the Enamine "antiviral-like" virtual library (the first column) are sorted by their total overlap score W_{All} (the second column). The values in the other columns correspond to the sums of the overlap scores of the conformers for the corresponding reference compounds.

**Compound ID**	**Wall**	**Olaparib**	**Tadalafil**	**Ergotamine**	**Remdesivir**
Z1693453146	254.11	71.68	56.30	76.76	49.38
Z434669842	248.84	66.18	55.33	69.92	57.41
Z1381427631	248.57	66.63	52.86	72.84	56.24
Z1381425049	247.97	66.09	53.03	71.98	56.88
Z1313285936	246.97	67.70	55.51	72.23	51.54
Z826278840	246.37	65.61	56.67	68.79	55.30
Z94559538	245.69	70.41	55.41	65.24	54.64
Z435640438	245.21	63.85	54.62	72.45	54.29
Z435642248	245.13	64.04	55.31	71.85	53.93
Z827564114	244.89	64.93	57.02	70.19	52.75

Tab. S2. The top scoring query compounds from the Enamine a "Diverse Discovery Set" virtual library (the first column) are sorted by their total overlap score W_{All} (the second column). The values in the other columns correspond to the sums of the overlap scores of the conformers for the corresponding reference compounds.

Compound ID	Wall	Olaparib	Tadalafil	Ergotamine	Remdesivir
Z1760146546	255.19	74.41	60.57	74.94	45.27
Z3077896041	254.26	67.67	57.38	75.53	53.67
Z2911083836	253.23	72.49	58.63	75.85	46.27
Z2446617864	252.49	73.18	59.52	75.06	44.72
Z1139281415	252.30	69.01	57.39	75.88	50.02
Z1354703942	251.79	77.22	61.63	80.63	32.32
Z2256366543	251.27	66.54	54.27	74.18	56.28
Z1139281396	250.99	68.40	57.90	74.43	50.27
Z1139280685	250.51	68.34	57.88	74.50	49.79
Z2959367287	250.32	69.54	55.40	72.80	52.58
